# Ten-Year Trends in Coronary Calcification in Individuals without Clinical Cardiovascular Disease in the Multi-Ethnic Study of Atherosclerosis

**DOI:** 10.1371/journal.pone.0094916

**Published:** 2014-04-17

**Authors:** Diane E. Bild, Robyn McClelland, Joel D. Kaufman, Roger Blumenthal, Gregory L. Burke, J. Jeffrey Carr, Wendy S. Post, Thomas C. Register, Steven Shea, Moyses Szklo

**Affiliations:** 1 Division of Cardiovascular Sciences, NHLBI, Bethesda, Maryland, United States of America; 2 Department of Biostatistics, University of Washington, Seattle, Washington, United States of America; 3 Department of Environmental and Occupational Health Sciences, Medicine, and Epidemiology, University of Washington, Seattle, Washington, United States of America; 4 Department of Medicine, Johns Hopkins University School of Medicine, Baltimore, Maryland, United States of America; 5 Department of Epidemiology, Johns Hopkins University, Bloomberg School of Public Health, Baltimore, Maryland, United States of America; 6 Wake Forest School of Medicine, Winston-Salem, North Carolina, United States of America; 7 Vanderbilt University School of Medicine, Nashville, Tennessee, United States of America; 8 Columbia University, New York, New York, United States of America; University of Groningen, Netherlands

## Abstract

**Background:**

Coronary heart disease (CHD) incidence has declined significantly in the US, as have levels of major coronary risk factors, including LDL-cholesterol, hypertension and smoking, but whether trends in subclinical atherosclerosis mirror these trends is not known.

**Methods and Findings:**

To describe recent secular trends in subclinical atherosclerosis as measured by serial evaluations of coronary artery calcification (CAC) prevalence in a population over 10 years, we measured CAC using computed tomography (CT) and CHD risk factors in five serial cross-sectional samples of men and women from four race/ethnic groups, aged 55–84 and without clinical cardiovascular disease, who were members of Multi-Ethnic Study of Atherosclerosis (MESA) cohort from 2000 to 2012. Sample sizes ranged from 1062 to 4837. After adjusting for age, gender, and CT scanner, the prevalence of CAC increased across exams among African Americans, whose prevalence of CAC was 52.4% in 2000–02, 50.4% in 2003–04, 60.0% is 2005–06, 57.4% in 2007–08, and 61.3% in 2010–12 (p for trend <0.001). The trend was strongest among African Americans aged 55–64 [prevalence ratio for 2010–12 vs. 2000–02, 1.59 (95% confidence interval 1.06, 2.39); p = 0.005 for trend across exams]. There were no consistent trends in any other ethnic group. Risk factors generally improved in the cohort, and adjustment for risk factors did not change trends in CAC prevalence.

**Conclusions:**

There was a significant secular trend towards increased prevalence of CAC over 10 years among African Americans and no change in three other ethnic groups. Trends did not reflect concurrent general improvement in risk factors. The trend towards a higher prevalence of CAC in African Americans suggests that CHD risk in this population is not improving relative to other groups.

## Introduction

Coronary heart disease mortality (CHD) has declined by more than 50% in the United States in the past four decades [1], [2], and the incidence [3] and prevalence [4] of CHD have also significantly declined in recent years. National trends in smoking, lipid levels [5], and hypertension control [6] have been favorable during this period, although rates of obesity and diabetes have risen^5^. The aggregate impact of these risk factor trends on atherosclerosis and risk for CHD is unclear. Data on subclinical atherosclerosis are not monitored, and it is not known if they mirror trends in CHD morbidity and mortality. Subclinical disease trends could inform our understanding of the relative impact of prevention and treatment and of the composite impact of CHD risk factors on CHD risk as well as help predict future clinical disease burden at the population level.

Coronary artery calcification (CAC) is a specific marker for atherosclerosis^7^ that reflects the extent of atherosclerotic plaque^8, 9^ and predicts CHD risk^10, 11^. Thus, the prevalence and extent of CAC would be expected to track with the burden of subclinical atherosclerotic disease and serve as an indicator of population risk for CHD events.

**Figure 1:**
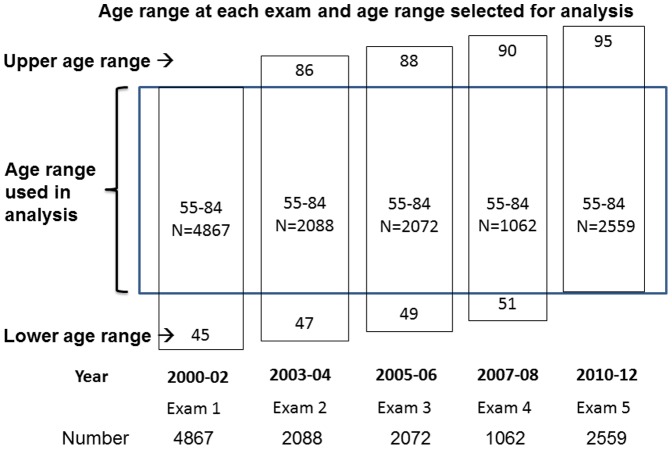
Participant selection. Participants whose ages fell between 55 and 84 years were selected from each examination. Note that many of the same individuals were thus included in multiple analyses but treated as if there were independent cross-sectional samples. Adjustment for age and use of robust standard errors were used to control for age and account for individuals being used multiple times in the analysis.

We examined the prevalence of CAC in five serial cross-sectional samples of men and women each aged 55–84 years from the cohort of the Multi-Ethnic Study of Atherosclerosis who were examined over a 10-year period. More specifically, we compared 55–84 year olds in 2000–02 to 55–84 year olds at four additional time points up to 2010–2012 (see Figure 1). Our hypotheses were that (1) the prevalence of CAC would decline over 10 years and (2) differences in risk factor levels and medication usage, particularly lipid-lowering therapy, would largely explain any decline observed.

## Methods

### Design, Setting, and Participants

Details of the study design have been published elsewhere [7]. In brief, between July 2000 and August 2002, 6,814 men and women who identified themselves as either White, African-American, Hispanic, or Chinese and were 45 to 84 years old and free of clinically apparent cardiovascular disease, were recruited from portions of six U.S. communities: Baltimore City and Baltimore County, MD; Chicago, IL; Forsyth County, NC; Los Angeles County, CA; Northern Manhattan and the Bronx, NY; and St. Paul, MN. Each field site recruited from locally available sources, including lists of residents, lists of dwellings, and telephone exchanges. In the last few months of the recruitment period, supplemental sources included lists of Medicare beneficiaries from the Centers for Medicare and Medicaid Services and referrals by participants to ensure adequate numbers of minorities and elderly subjects. The study was approved by the institutional review boards at all participating centers, including the Johns Hopkins University School of Medicine Joint Committee on Clinical Investigation, Harbor-UCLA Research and Education Institute Human Subjects Committee, University of Vermont Committees on Human Research, University of Minnesota Human Research Protection Program, UCLA Office of Human Research Protection Program, Northwestern University Social and Behavioral Sciences Institutional Review Board, University of Washington Human Subjects Division, Wake Forest University Health Sciences Office of Research Institutional Review Board, and Columbia University Medical Center Institutional Review Board, and all participants gave written informed consent. Participants were invited back for 4 subsequent examinations. Approximately half of the returning cohort had repeat CT scans during 2002–03, with the other half scanned during 2004–05, approximately 25% were rescanned during 2007–08, and ∼70% were selected for rescanning during 2010–12 based on prior participation in a substudy [8]. Participants were not selected for rescanning based on any clinical characteristics and were thus treated as random samples.

We included participants aged 55–84 at each exam who had a CT scan, excluding those who had reported having had cardiovascular disease prior to the exam. Persons with atrial fibrillation were also excluded.

### CT scanning

Computed tomography scanning of the chest was performed using either ECG-triggered (at 80% of the RR interval) electron-beam computed tomography scanner (Chicago, Los Angeles, and New York Field Centers) (Imatron C-150, Imatron, San Francisco) or using prospectively ECG- triggered scan acquisition at 50% of the RR interval with a multi-detector computed tomography system acquiring four simultaneous 2.5 mm slices for each cardiac cycle in a sequential or axial scan mode (Baltimore, Forsyth County and St. Paul Field Centers) (Lightspeed, General Electric, Waukesha, WI or Siemens, Volume Zoom, Erlangen, Germany). During a study, each participant was scanned twice except during Exam 5, when each participant received one cardiac scan. Scans were read centrally at the Harbor-University of California, Los Angeles Research and Education Institute, to identify and quantify coronary calcification. Calcium scores among scanning centers and between participants were adjusted using a standard calcium phantom scanned simultaneously with the participant. The average Agatston score was used in all analyses [9]. The presence of calcification was defined as an Agatston score >0 on any scan. Agreement regarding presence of coronary calcification was high (Kappa statistic 0.90 to 0.93 between and within readers), and the intraclass correlation coefficient for the Agatston score between readers was 0.99 [9].

### Assessment of Risk Factors

At each examination, standardized questionnaires were used to obtain information about level of education, annual household income, smoking history and medication usage for high blood pressure, high cholesterol or diabetes. Smoking was defined as current, former, or never. Height and weight were measured with light clothing and without shoes. Body mass index (BMI) was calculated as weight in kilograms divided by height in meters squared. Resting blood pressure was measured three times in the seated position using a Dinamap model Pro 100 automated oscillometric sphygmomanometer (Critikon, Tampa, FL). The average of the last two measurements was used in analysis. Hypertension was defined as systolic pressure greater than or equal to 140 mm Hg, diastolic pressure greater than or equal to 90 mm Hg or current use of anti-hypertensive medication. Total and HDL cholesterol, triglycerides, and glucose were measured from blood samples obtained after a 12-hour fast. LDL-cholesterol was calculated using the Friedewald equation. Diabetes was defined as fasting glucose greater than 6.99 mmol/L (126 mg/dl) or current use of hypoglycemic medication. We calculated a Framingham Risk Score (FRS) for CHD for each participant [10].

### Statistical Analysis

We examined risk factor levels and prevalence at each exam but for simplicity report levels at baseline and year 10, as they reflect overall trends. The prevalence of CAC at each exam was estimated using the following cutpoints of the Agatston score, after adjusting for age, gender, ethnicity, and scanner: 0 vs. >0 and 0, 1–99, 100–299, and 300 or higher. We compared the prevalence of having any CAC using prevalence rate regression, adjusting for the distribution of age, gender, and scanner type from the baseline exam. The goal at each exam was to create comparable samples by applying the same exclusion criteria that were used for entry into MESA. Some participants “age in” at later exams (those who were younger than 55 at baseline), and some participants “age out” at later exams once they become 85 or older. Some participants are included in multiple exams; however, we were not interested in modeling change over time for particular participants. Cluster-robust standard errors were used to account for the fact that some participants will appear in multiple subsets. This allows for the fact that multiple observations from the same participant are not independent. Such dependence would not impact the coefficients/prevalence ratios, but would result in underestimated standard errors. The cluster-robust standard errors deal with this issue, with observations within a participant assumed to be correlated, and observations from different participants independent.

Differences in trends by ethnic group were noted, and further analyses were stratified by ethnicity. Additionally, we constructed a model that controlled for the use of lipid-lowering therapy, cholesterol levels, anti-hypertensive therapy, blood pressure levels, and smoking. We added an exam-by-lipid-lowering therapy interaction term and also applied the fully-adjusted model to participants who were not currently taking lipid-lowering medication. We examined models that included only non-overlapping samples (excluding those aged 60–69). Finally, analyses were stratified by age group and gender to determine if trends were similar among age and gender subgroups. Tests for trends in CAC prevalence over time were performed for each model.

## Results

There were 4837 and 2532 participants included at baseline and year 10, respectively, who were aged 55–84, without a history of cardiovascular disease and had complete data for analysis (Table 1). The mean age was 67.1 years at baseline and 68.0 years at year 10. The gender and ethnic distributions were similar. The proportion who did not complete high school was lower at year 10. The prevalence of current smoking was lower, while the prevalence of former smoking was higher. Average systolic and diastolic blood pressures were lower by 8.0 mmHg and 3.0 mmHg, respectively, while the proportion with hypertension increased from 52.9% to 55.6%, due to increased use of antihypertensive medication. Total cholesterol decreased 8.0 mg/dl, HDL-cholesterol increased by 4.4 mg/dl, and the proportion of participants who reported taking lipid-lowering medication increased from 19.7% to 35.0%. The prevalence of diabetes increased from 14.6% to 17.9%, although mean body mass index was only 0.4 units higher. The FRS decreased from 9.1% to 7.4%. The proportion with CAC increased from 60.0% to 65.8% (p<0.0001). Trends in risk factors and FRS were consistent across the ethnic groups (Table S1).

**Table 1:** Characteristics of the Cohorts at Baseline and Exam 5, Multi-Ethnic Study of Atherosclerosis, 2000–02 and 1010–12.

	Baseline	Exam 5	
	2000–02	2010–12	p-value
Number	4837	2532	—
Age (yrs)	67.1± 7.5	68.0 ± 8.1	<0.001
Gender (% male)	2291 (47.4)	1183 (46.7)	0.53
Race/ethnicity (%)			0.46
White	1887 (39.0)	974 (38.5)	
African American	1343 (27.8)	684 (27.0)	
Hispanic	1030 (21.3)	553 (21.8)	
Chinese	577 (11.9)	321 (12.7)	
Education <high school (%)	994 (20.5)	342 (13.5)	<0.001
Smoking status (%)			<0.001
current	518 (10.7)	210 (8.3)	
former	1908 (39.4)	1158 (45.7)	
never	2411 (49.8)	1164 (46.0)	
Systolic BP (mmHg)	131.0 ± 21.8	123.0 ± 20.3	<0.001
Diastolic BP (mmHg)	71.9± 10.2	68.9 ± 9.9	<0.001
Anti-hypertensive medications (%)	2126 (44.0)	1288 (50.9)	<0.001
Hypertension (%)	2561 (52.9)	1408 (55.6)	0.015
Total cholesterol (mg/dl)	194.0 ± 35.6	186.0 ± 36.0	<0.001
HDL Cholesterol (mg/dl)	51.5± 15.1	55.9+/16.7	<0.001
Lipid lowering medications (%)	953 (19.7)	886 (35.0)	<0.001
Body Mass Index (kg/m^2^)	28.2 ± 5.3	28.6± 5.5	0.001
Diabetes (%)	708 (14.6)	454 (17.9)	<0.001
FRS (10-year risk, %)	12.8 (9.1)	10.1(7.4)	<0.001
Prevalence of CAC (%)	2904 (60.0)	1665 (65.8)	<0.001

Data are mean ± standard deviations or number (%). P-values are based on regression models (linear or logistic) with cluster-robust standard errors. BP = blood pressure; FRS = Framingham Risk Score; CAC = coronary artery calcification.

Of the 8 groups of scanners employed in the study over 10 years, one scanner used only during the first three exams was associated with a 5.5% higher prevalence of CAC (p = 0.008) (data not shown).

**Figure 2:**
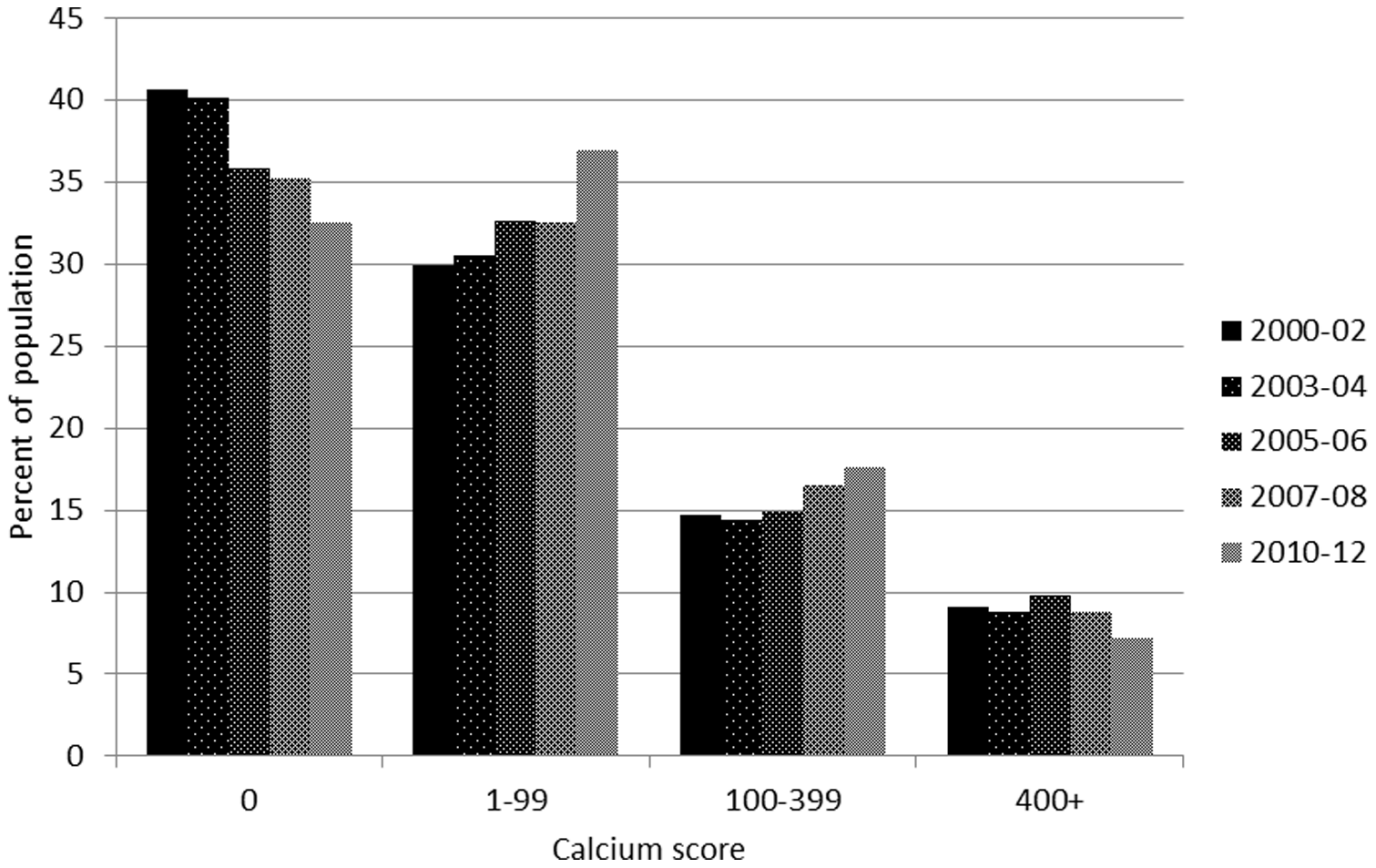
Adjusted prevalence of CAC at different levels among those aged 55–84 across exams, Multi-Ethnic Study of Atherosclerosis. Adjusted to the average baseline age (67 years), gender (47% male), race/ethnicity (39% white, 28% African American, 21% Hispanic, and 12% Chinese), and scanner (electron-beam CT vs. other).

After adjusting for age, gender, ethnicity, and scanner, the proportion of participants with no CAC decreased over time from 40.7% to 32.6% (p = 0.007), and the proportions with a CAC score 1–99 increased from 29.9% to 37.0% (p = 0.01), with a CAC score of 100–299 increased from 14.7% to 17.7% (p = 0.14), and with CAC score 400 or over decreased from 9.1% to 7.2% (p = 0.11) (Figure 2). Comparing Exam 5 (2010–12) to baseline (2000–02) the adjusted prevalence ratio for CAC >0 was 1.08 (p<0.001 for trend).

Trends in CAC among the four race/ethnic groups revealed a significant trend towards increased prevalence of CAC in African Americans but not in any other group (Table 2); the p-value for a differences in trends in African Americans compared to the other groups after adjusting for all other variables was 0.049. Among African Americans, the CAC prevalence ratio (year 10 vs. baseline) was 1.27 (p<0.001 for test for trend). Adjustment for risk factors made no notable difference in CAC trends in any ethnic group. Similar trends were found when the definition of CAC prevalence was changed from an Agatston score >0 to an Agatston score >10 (Table S2).

**Table 2:** Prevalence of CAC>0 at baseline and Year 10 and relative prevalence of CAC>0 across exams among those aged 55–84 years without cardiovascular disease, 2000–02 through 2010–12, by ethnicity, Multi-Ethnic Study of Atherosclerosis.

	Exam	White	African American	Hispanic	Chinese
Baseline CAC prevalence		67.50%	52.20%	56.60%	59.80%
Year 10 CAC prevalence		71.50%	61.30%	62.40%	63.90%
Adjusted for age, gender, and scanner	Baseline	Ref 95% CI	Ref 95% CI	Ref 95% CI	Ref 95% CI
	2	1.00 [0.97,1.04]	0.96 [0.91,1.02]	1.02 [0.96,1.08]	1.00 [0.93,1.08]
	3	1.02 [0.98,1.06]	1.14 [1.07,1.22]	1.09 [1.00,1.18]	1.01 [0.93,1.09]
	4	1.03 [0.96,1.10]	1.14 [1.04,1.26]	1.11 [1.00,1.24]	0.97 [0.85,1.11]
	5	1.01 [0.92,1.11]	1.27 [1.11,1.47]	1.14 [0.97,1.34]	1.06 [0.98,1.15]
p-value for trend		0.64	0.001	0.15	0.73
Adjusted for age, gender, education, scanner, and risk factors	Baseline	Ref 95% CI	Ref 95% CI	Ref 95% CI	Ref 95% CI
	2	1.01 [0.97,1.04]	0.99 [0.93,1.05]	1.00 [0.95,1.06]	1.00 [0.92,1.08]
	3	1.01 [0.97,1.05]	1.13 [1.06,1.21]	1.09 [1.00,1.18]	1.03 [0.95,1.11]
	4	1.03 [0.97,1.10]	1.15 [1.04,1.26]	1.11 [1.00,1.23]	0.97 [0.85,1.11]
	5	1.01 [0.92,1.11]	1.26 [1.10,1.44]	1.11 [0.95,1.29]	1.06 [0.97,1.16]
p-value for trend		0.67	0.001	0.24	0.94

Risk factors included total and HDL cholesterol, lipid-lowering medication, systolic blood pressure, anti-hypertensive medication, presence of diabetes, and smoking status (current, former, never). The p-value testing for differences in trends was p = 0.10 adjusting for age, gender and scanner, and p = 0.14 adjusting for age, gender, scanner, and risk factors. Tests for differences in trends in African Americans compared to the other three groups was p = 0.029 and p = 0.049 respectively.

Trends towards higher CAC prevalence were further concentrated among younger African Americans (Table 3). Adjusted prevalence ratios were 1.67 among African Americans aged 55–64 (p = 0.005 for trend across exams), 1.39 among African Americans aged 65–74, and 0.92 among African Americans aged 75–84 (p = 0.54 for trend across exams). Only among Chinese aged 65–74 was there a similar and significant, but more modest trend, towards increased CAC: prevalence ratio 1.12 (p = 0.03 for trend across exams).

**Table 3:** Prevalence of CAC>0 at baseline and Year 10 and relative prevalence of CAC>0 across exams among those aged 55–84 years without cardiovascular disease, 2000–02 through 2010–12, by ethnicity, Multi-Ethnic Study of Atherosclerosis.

	Exam	White	African American	Hispanic	Chinese
Baseline CAC prevalence		51.30%	37.40%	41.50%	48.20%
Age 55–64	Baseline	Ref [95% CI]	Ref [95% CI]	Ref [95% CI]	Ref [95% CI]
	2	1.06 [0.97,1.16]	1.03 [0.89,1.18]	1.00 [0.87,1.15]	1.08 [0.91,1.30]
	3	1.08 [0.99,1.18]	1.26 [1.07,1.48]	1.23 [1.00,1.51]	0.88 [0.70,1.10]
	4	1.10 [0.95,1.27]	1.51 [1.18,1.93]	1.26 [0.93,1.70]	0.76 [0.47,1.25]
	5	1.13 [0.88,1.45]	1.67 [1.10,2.51]	1.10 [0.73,1.66]	0.96 [0.77,1.19]
p-value for trend		0.16	0.005	0.85	0.08
Baseline CAC prevalence		72.10%	54.00%	62.30%	63.60%
Age 65–74	Baseline	Ref [95% CI]	Ref [95% CI]	Ref [95% CI]	Ref [95% CI]
	2	1.01 [0.96,1.07]	0.97 [0.87,1.07]	1.02 [0.94,1.12]	0.97 [0.85,1.10]
	3	0.98 [0.91,1.05]	1.20 [1.08,1.33]	1.09 [0.96,1.24]	1.1 [0.98,1.24]
	4	0.99 [0.89,1.09]	1.08 [0.92,1.28]	1.13 [0.95,1.35]	1.17 [0.97,1.41]
	5	0.88 [0.77,1.01]	1.39 [1.11,1.74]	1.19 [0.92,1.53]	1.12 [0.95,1.31]
p-value for trend		0.1	0.005	0.19	0.03
Baseline CAC prevalence		87.70%	78.30%	77.60%	73.60%
Age 75–84	Baseline	Ref [95% CI]	Ref [95% CI]	Ref [95% CI]	Ref [95% CI]
	2	0.98 [0.93,1.03]	1.00 [0.92,1.09]	0.98 [0.89,1.07]	1.03 [0.91,1.17]
	3	0.98 [0.93,1.03]	1.00 [0.90,1.11]	1.05 [0.92,1.21]	1.07 [0.95,1.22]
	4	1.02 [0.91,1.14]	1.05 [0.92,1.20]	1.06 [0.92,1.23]	1.02 [0.84,1.22]
	5	1.08 [0.94,1.25]	0.92 [0.75,1.13]	1.09 [0.88,1.34]	1.15 [1.00,1.32]
p-value for trend		0.31	0.54	0.48	0.63

Adjusted for age, gender, education, scanner, and risk factors, including total and HDL cholesterol, lipid-lowering medication, systolic blood pressure, anti-hypertensive medication, presence of diabetes, and smoking status (current, former, never).

There were no significant differences in these trends by gender (data not shown). Excluding data obtained from the scanner associated with higher CAC, restricting the analysis to those not on lipid-lowering medications, and excluding participants aged 55–74 at baseline who would otherwise be included at both baseline and Year 10 did not affect findings (data not shown).

## Discussion

We found secular trends towards a higher prevalence of CAC over a 10-year period among African American men and women who did not have clinical cardiovascular disease, particularly among those aged 55–74 years. We found no consistent, statistically significant trends in any other ethnic group. This finding was contrary to our hypothesis that the secular trends in CAC would parallel recent downward trends in cardiovascular mortality and incidence, and it was not consistent with the concomitant improvement in Framingham Risk Score. However, it may be consistent with evidence of differential trends in CHD risk by ethnicity. For example, the proportion of Americans with at least one of three major coronary risk factors decreased significantly between 1999–2000 and 2009–2010 in the National Health and Nutrition Examination Surveys among non-Hispanic white and Mexican-American adults but not among non-Hispanic blacks [11]. Also, the Atherosclerosis Risk in Communities Study found steeper declines in incidence of myocardial infarction in whites than African Americans between 1987 and 2008 [12]. We explored several explanations for these trends, including increased sensitivity of CT scanners time, use of statin therapy, which might artifactually increase CAC scores, and an increase in the prevalence of diabetes. However, none of these explanations appears to explain the increase in African Americans or to modify the trend in any other group.

Coronary calcification is a specific marker for atherosclerosis [13]–[15], although it is not sensitive to non-calcified plaque, and its presence may represent other, non-atherosclerotic processes [16]. Still, because of its close pathologic ties to atherosclerotic plaque and its ability to predict cardiovascular events [17], [18], it may be considered a marker of both individual and population risk of cardiovascular disease, particularly CHD. The trend towards an increased prevalence of CAC among African Americans is consistent with other data suggesting that the favorable trends in CHD experienced in the general population is not being paralleled in this group [11], [12]. _ENREF_10

We used a longitudinal cohort study to assess secular trends, a method that has been employed in previous population-based longitudinal studies [19]–[21]. We found trends in risk factors that are similar to trends using more conventional independent cross-sectional samples, such as from NHANES [5]. This includes declines in smoking [22], systolic and diastolic blood pressure, total cholesterol [23], and increases in HDL-cholesterol [23], the use of antihypertensive medication [6], use of lipid-lowering medication, body mass index [22], and diabetes. Lower socioeconomic status has been associated with higher CAC prevalence [24] though not consistently [25]. However, adjustment for trends in these variables did not alter the trend in CAC in any group.

Our study has several limitations. The main limitation is that our sampling design attempted to replicate independent serial cross-sectional samples by our sampling design and statistical adjustments, but we may not have completely eliminated selection bias stemming from participation in a longitudinal study. For example, the significant decrease in the proportion with no more than high school education serves as an indication that less well educated participants tended not to return and were therefore less well represented groups after baseline. We adjusted for education and other potential confounders to mitigate against these potential biases. In addition, the samples were not necessarily representative of the US population and, in particular, could not include in-migration of groups that would be expected to affect population risk. We assumed that this effect would be small. Finally, the samples were not independent; however, we did also account for this in the statistical analysis, including an analysis of participants who did not overlap between baseline and year 10.

African Americans, the group that displayed a significant trend towards a higher prevalence of CAC, also had the lowest CAC prevalence at baseline, raising the question of whether this group had a higher “ceiling” than the others, with more opportunity to experience an upward trend. We do not believe that the secular trends would differ based on initial CAC prevalence, particularly given that no group was close to zero prevalence or 100% prevalence, and therefore, with no opportunity to decrease or increase, respectively, but it remains a possible explanation for the differences observed among the ethnic groups.

The changes in CT scanner technology over the 10 years could have contributed artifact to trends in CAC. The study began using three types of scanners in six centers; over 10 years, 12 different scanners were employed. While the same scoring system was used over time, and scores were adjusted using a calcium phantom, the potential exists that newer models of scanners tended to identify more calcification. We did determine that one scanner was associated with a higher prevalence of CAC, but this did not explain the trends because it was used only during the first 3 exams, its use was not predominant among sites with African Americans, and excluding this scanner did not change the observed trends in CAC.

Another factor that could have potentially modified CAC prevalence was the dramatic increase in the use of statins. Statins lower serum LDL-cholesterol and have been shown to be associated with lower fibrofatty plaque volume [26] and regression of atherosclerosis [27] and either no change [28] or even an increase in the relative amount of calcium, [29] a phenomenon posited to be due to an increase in the concentration of calcium within plaques. (Statins comprised 93–95% of the lipid-lowering medications used across the exams in MESA.) In a recent study of patients who received either pitavastatin or pravastatin, intravascular ultrasound measurements for 119 patients before and after 8 months of treatment showed significant increases in the amount of calcium imaged – from 0.42 to 0.55 mm ^3^/mm and 0.44 to 0.55 mm ^3^/mm, respectively, for two statin treatments [29]. The Agatston score is created using a step function that gives increasing weight to foci with greater Hounsfield units, and it is thus possible that plaques with denser lesions due to statin therapy could have higher Agatston scores.

A final limitation of note is our inability to confirm the trends in CAC prevalence with trends in CHD incidence due to limited follow-up duration, specifically for the latter years of the study.

The concentration of a trend towards increased CAC among African Americans aged 55–64 and, to a lesser extent, aged 65–74, is notable. While cardiovascular risk factors have generally improved in this population and in the general population, obesity and diabetes have increased. Diabetes is strongly associated with CAC [30], [31]_ENREF_25 and with increased CHD risk [32], [33]. However, the African Americans in this cohort, including among those aged 55–64, in whom CAC trends were strongest, did not exhibit stronger trends toward obesity and diabetes than other groups (see Table S1). At baseline in MESA, and in most other studies comparing ethnic groups, African Americans have a significantly lower prevalence of CAC than whites [25]. Despite these trends, the relative ranking of prevalence of CAC after 10 years among the four ethnic groups remained the same, with African Americans having the lowest, and whites having the highest prevalence.

The trends in CAC were unrelated to trends in CHD risk factors over ten years; thus, CAC prevalence may not be a good indicator of ten year temporal changes in CHD risk in a population. Because coronary atherosclerosis is known to initiate in early in life and progress over the decades of early adult life, CAC likely represents lifelong cumulative exposure to risk factors, and thus changes in exposure to risk factors over a relatively short period (10 years, in this case) may not significantly affect CAC prevalence or the presence of coronary atherosclerosis.

In summary, we found an increasing prevalence of CAC over a 10-year period among middle-aged African Americans, but not in whites, Hispanics, or Chinese with no history of clinical cardiovascular disease. Trends in CAC were unrelated to trends in risk factors over this period.

## Supporting Information

Table S1 Characteristics of the Cohorts at Baseline and Exam 5 by Race/Ethnicity, Multi-Ethnic Study of Atherosclerosis, 2000–02 and 2010–12.
(DOCX)

Table S2 Prevalence of CAC>10 at baseline and Year 10 and relative prevalence of CAC>10 across exams among those aged 55–84 years without cardiovascular disease, 2000–02 through 2010–12, by ethnicity, Multi-Ethnic Study of Atherosclerosis.
(DOCX)
