# Correction: Ten-Year Trends in Coronary Calcification in Individuals without Clinical Cardiovascular Disease in the Multi-Ethnic Study of Atherosclerosis

**DOI:** 10.1371/journal.pone.0103666

**Published:** 2014-07-21

**Authors:** The PLOS ONE Staff

The following information is missing from the Funding section: This research was supported by contracts N01-HC-95159, N01-HC-95160, N01-HC-95161, N01-HC-95162, N01-HC-95163, N01-HC-95164, N01-HC-95165, N01-HC-95166, and N01-HC-95169 from the National Heart, Lung, and Blood Institute, by grants UL1-TR-000040 and UL1-RR-025005 from NCRR, and by grant RD831697 (MESA Air) from the U.S Environmental Protection Agency.
