# Supplementary material for: Ten-Year Trends in Coronary Calcification in Individuals without Clinical Cardiovascular Disease in the Multi-Ethnic Study of Atherosclerosis
Source: PLoS One. 2014 Apr 17;9(4):e94916. doi: 10.1371/journal.pone.0094916 (PMC3990562; doi:10.1371/journal.pone.0094916)
Supplement: Table S1 — Characteristics of the Cohorts at Baseline and Exam 5 by Race/Ethnicity, Multi-Ethnic Study of Atherosclerosis, 2000–02 and 2010–12. (DOCX) [file pone.0094916.s001.docx]

Table S1

| \| Prevalence of CAC>10 at baseline and Year 10 and relative prevalence of CAC>10 across exams among those aged 55-84 years without cardiovascular disease, 2000-02 through 2010-12, by ethnicity, Multi-Ethnic Study of Atherosclerosis \| \| --- \| | | | | | | |
| --- | --- | --- | --- | --- | --- | --- | --- |
|  | Exam | White | African American | Hispanic | Chinese | |
| Baseline CAC>10 prevalence |  | 60.1% | 45.3% | 49.6% | 51.5% | |
| Year 10 CAC>10 prevalence |  | 64.5% | 52.6% | 53.3% | 56.8% | |
| Adjusted for age, gender, and scanner | Baseline  2  3  4  5 | Ref 95% CI  1.01 [0.97,1.05]  1.00 [0.96,1.05]  1.01 [0.93,1.09]  1.01 [0.90,1.14] | Ref 95% CI  0.95 [0.89,1.02]  1.06 [0.98,1.15]  1.19 [1.06,1.34]  1.24 [1.04,1.47] | Ref 95% CI  1.02 [0.96,1.09]  0.98 [0.88,1.09]  1.02 [0.88,1.17]  1.03 [0.84,1.27] | Ref 95% CI  1.04 [0.96,1.14]  1.01 [0.92,1.11]  1.01 [0.86,1.17]  1.09 [0.99,1.20] |  |
| p-value for trend |  | 0.81 | 0.010 | 0.72 | 0.96 | |
| Adjusted for age, gender, education, scanner, and risk factors | Baseline  2  3  4  5 | Ref 95% CI  1.01 [0.97,1.05]  1.00 [0.95,1.05]  1.01 [0.94,1.09]  1.01 [0.91,1.13] | Ref 95% CI  0.97 [0.90,1.04]  1.04 [0.96,1.13]  1.17 [1.05,1.32]  1.23 [1.04,1.45] | Ref 95% CI  1.00 [0.94,1.07]  0.99 [0.89,1.10]  1.04 [0.92,1.19]  1.02 [0.84,1.24] | Ref 95% CI  1.03 [0.94,1.13]  1.03 [0.94,1.13]  0.99 [0.84,1.17]  1.09 [0.98,1.21] | |
| p-value for trend |  | 0.81 | 0.012 | 0.78 | 0.87 | |

Risk factors included total and HDL cholesterol, lipid-lowering medication, systolic blood pressure, anti-hypertensive medication, presence of diabetes, and smoking status (current, former, never).
